# Dissociating Long and Short-term Memory in Three-Month-Old Infants Using the Mismatch Response to Voice Stimuli

**DOI:** 10.3389/fpsyg.2018.00031

**Published:** 2018-01-30

**Authors:** Katharina Zinke, Leonie Thöne, Elaina M. Bolinger, Jan Born

**Affiliations:** ^1^Institute of Medical Psychology and Behavioral Neurobiology, University of Tübingen, Tübingen, Germany; ^2^Werner Reichardt Center for Integrative Neuroscience, University of Tübingen, Tübingen, Germany

**Keywords:** ERPs, early infancy, familiarity, voice discrimination, long-term memory, MMN

## Abstract

Auditory event-related potentials (ERPs) have been successfully used in adults as well as in newborns to discriminate recall of longer-term and shorter-term memories. Specifically the Mismatch Response (MMR) to deviant stimuli of an oddball paradigm is larger if the deviant stimuli are highly familiar (i.e., retrieved from long-term memory) than if they are unfamiliar, representing an immediate change to the standard stimuli kept in short-term memory. Here, we aimed to extend previous findings indicating a differential MMR to familiar and unfamiliar deviants in newborns (Beauchemin et al., 2011), to 3-month-old infants who are starting to interact more with their social surroundings supposedly based on forming more (social) long-term representations. Using a voice discrimination paradigm, each infant was repeatedly presented with the word “baby” (400 ms, interstimulus interval: 600 ms, 10 min overall duration) pronounced by three different female speakers. One voice that was unfamiliar to the infants served as the frequently presented “standard” stimulus, whereas another unfamiliar voice served as the “unfamiliar deviant” stimulus, and the voice of the infant’s mother served as the “familiar deviant.” Data collection was successful for 31 infants (mean age = 100 days). The MMR was determined by the difference between the ERP to standard stimuli and the ERP to the unfamiliar and familiar deviant, respectively. The MMR to the familiar deviant (mother’s voice) was larger, i.e., more positive, than that to the unfamiliar deviant between 100 and 400 ms post-stimulus over the frontal and central cortex. However, a genuine MMR differentiating, as a positive deflection, between ERPs to familiar deviants and standard stimuli was only found in the 300–400 ms interval. On the other hand, a genuine MMR differentiating, as a negative deflection, between ERPs to unfamiliar deviants from ERPs to standard stimuli was revealed for the 200–300 ms post-stimulus interval. Overall results confirm a differential MMR response to unfamiliar and familiar deviants in 3-month-olds, with the earlier negative MMR to unfamiliar deviants likely reflecting change detection based on comparison processes in short-term memory, and the later positive MMR to familiar deviants reflecting subsequent long-term memory-based processing of stimulus relevance.

## Introduction

Our capacity to effectively interact with our environment relies on our ability to utilize both short-term and long-term memory (Atkinson and Shiffrin, 1968). Short-term memory allows us to process and evaluate the constant stream of information (e.g., sensory input) that we are constantly confronted with when awake. It holds information for a short time period and thereby enables us to filter out important information, e.g., by recognizing if a sound is different, and therefore possibly threatening, from the stream of environmental sounds (so-called change detection). Long-term memory representations, on the other hand, are representations of events or information that we have encountered before and are stored for a longer time (hours to years), usually because of their relevance in guiding behavior (either due to emotional relevance or because they represent environmental regularities). These long-term representations can bias short-term processes by drawing attention to relevant items being held in short-term memory. Recognition processes, especially in the auditory domain, start to develop early during ontogeny (Fagan, 1973; Pascalis and de Haan, 2003; Jabès and Nelson, 2015) possibly connected to a relatively early maturation of several brain regions that are known to subserve memory formation such as the hippocampus (Seress et al., 2001; Huber and Born, 2014). For example, newborns and even fetuses show a preference for their own mother’s voice compared to the voice of a stranger (DeCasper and Fifer, 1980; Lee and Kisilevsky, 2014). This suggests that infants form long-term representations of familiar voices and use them to guide the short-term processing of incoming auditory information (like environmental sounds, voices, etc.) to identify their mother.

Event-related electrical brain potentials (event-related potentials, ERPs) have been successfully used in adults and newborns to quantify and dissociate auditory short-term and long-term memory. One of the earliest change detection responses reliably induced by any discriminable change in auditory stimulation is the mismatch negativity (MMN) which is frequently termed Mismatch Response (MMR) in the developmental literature because it can change in polarity in infants (MMR: Cheour-Luhtanen et al., 1996; Cheour et al., 2000; Leppänen et al., 2004; Näätänen et al., 2007). The MMR is thought to reflect a comparison process in short-term memory between the sensory memory traces of a repeated presentation of a (standard) stimulus and the neural trace of an infrequent stimulus (a so-called deviant; Cheour et al., 2000; Näätänen et al., 2007). Interestingly, MMR amplitude, latency, and duration are modulated by familiarity of the deviant stimulus, making it possible to dissociate influences of longer-term memory processes in change detection as well. For example, native language phonemes evoke larger MMRs after fewer repetitions of the standard stimuli than foreign language phonemes (in adults, Näätänen et al., 1997; Huotilainen et al., 2001; and infants, Cheour et al., 1998) and training of auditory discrimination increases the MMR for practiced stimuli (in adults, Atienza and Cantero, 2001; Tervaniemi et al., 2001; Näätänen et al., 2007; and newborns, Cheour et al., 2002; Partanen et al., 2013). These modulations of the MMR are thought to reflect long-term memory-based processing of relevance that occurs in addition to the short-term memory-based change detection processes.

In newborns, a larger, more positive MMR is elicited by a familiar deviant (the voice of the infant’s mother) compared to an unfamiliar one (Beauchemin et al., 2011). Interestingly, in that study, both the unfamiliar and the familiar deviant voice elicited an MMR of positive polarity, although the MMR to the familiar deviant occurred earlier and exhibited a larger amplitude than the MMR to the unfamiliar deviant. This suggests that the newborn brain uses similar mechanisms for detecting deviation regardless of whether the deviant stimulus is familiar (and represented in long-term memory) or unfamiliar (with only a short-term representation available). Here, we aimed to extend the findings by Beauchemin et al. (2011) of a modulation of the MMR depending on the familiarity of the deviant to 3-month-old infants (10–18 weeks) who have gained a vast amount of experience with voices and are in a window of unique plasticity in the auditory cortex (synaptogenesis reaches a peak at 3 months, Huttenlocher and Dabholkar, 1997). At this particular age infants are also starting to interact more with their social surroundings supposedly based on the formation of social long-term representations (e.g., the emergence of a social smile). We were particularly interested in whether and to what extent at this age long-term memory-based and short-term memory-based processing of familiar and unfamiliar deviant stimuli, respectively, would already express itself in more dissociable MMR features. Specifically, in addition to a larger, more positive MMR to a familiar deviant reflecting long-term memory-based processing of deviation, we expected to find signs of a negative MMR to unfamiliar deviants, reminiscent of the emergence of a robust MMN during the first post-natal months characterizing short-term memory-based processing of deviation (Kushnerenko et al., 2002; Jing and Benasich, 2006; He et al., 2009).

## Materials and Methods

### Participants

Overall, the data of 31 infants (15 females) between 10 and 18 weeks (99.7 ± 2.9 days; range: 74–130 days) were included in analyses. All infants were born singleton at full-term (mean gestational age: 39.8 ± 0.2 weeks) with normal neonatal outcome (birth weight > 2,500 g, mean birth weight: 3,524 ± 83.0 g, mean birth height 51.7 ± 0.4 cm), were healthy according to parental report, and had no severe complications during pregnancy or delivery. All infants had an Apgar score above 9 at 10 min after birth (median 9/10/10 for the 1/5/10 min Apgar score). All infants were breastfeed, some of them partially substituted by formula (*n* = 4). Three additional infants were tested but excluded due to excessive artifacts or extreme fussiness. Parents of participants were recruited via email advertisements across the universities mailing system, flyers, and through mothers who had already participated in another study during pregnancy with their child. Participating families received monetary compensation for their time and effort. The study was part of a larger study focusing on the role of sleep for memory processing in infants and was approved by the ethics committee of the Medical Faculty of the University and University Clinics Tübingen.

### Voices Oddball Task

The oddball paradigm was adapted from a previous study investigating the discrimination of a familiar from an unfamiliar voice in newborns (Beauchemin et al., 2011). Instead of using a single vowel we chose to use the word “baby” as the target stimulus (like in other studies exploring the processing of the mother’s voice in infants, e.g., deRegnier et al., 2000; Mai et al., 2012) because it contains richer acoustic features possibly related to voice discrimination processes than that of a short snippet of a vowel. For each infant, the paradigm consisted of repeatedly presented recordings of the word “baby” (400 ms, ISI = 600 ms) pronounced by three different female speakers: an unfamiliar one (frequently presented “standard” stimulus, 85% of the trials, *n* = 510), the infants’ own mother (infrequently presented “familiar deviant,” 7.5% of the trials, *n* = 45), and a second unfamiliar one (“unfamiliar deviant,” 7.5% of the trials, *n* = 45). The standard and the unfamiliar deviant stimuli for each infant were chosen from a pool of four female voice recordings – allocation of the voices was balanced across participants. Stimuli were presented in a pseudorandomized order using the software Presentation (Neurobehavioral Systems©, Berkeley, CA, United States) with the constraint that every presentation of a deviant had to be followed by at least three standard stimuli in order to facilitate a robust MMR response (Cheour et al., 2000; Beauchemin et al., 2011). Stimulus presentation lasted for a total of 10 min (600 trials in total) and was played to the awake infants. The stimuli were presented binaurally through loudspeakers at a constant sound pressure peaking at ∼75 dB to avoid differences in ERPs elicited by differences in intensity of the stimuli. For recording the task stimuli, infants’ mothers and the four other female speakers were instructed to pronounce the word “baby” with a German pronunciation as naturally as possible while avoiding any emotional connotations. Thus, voice recordings represent instances of non-infant directed speech. The voices were recorded with a portable USB Condenser Microphone (Go Mic by Samson Technologies^®^) and custom-made pop filter using the software Audacity 2.0.5 for recording and post-processing. Minimal processing was necessary to produce stimuli of comparable length and loudness using noise removal, amplifying, cutting recordings and changing tempo minimally where necessary.

### Sleepiness and Control Variables

To control for possible effects of sleepiness on acute attentional and memory processes, sleep duration in the last 24 h was assessed by asking the mothers (“When and for how long did your child sleep within the last 24 h?”). Additionally, in a subsample of 23 infants, the mothers were asked to judge the infants’ level of sleepiness on a 10-point scale (from 1, “very awake,” to 10, “asleep”) right before starting the voice paradigm.

### Procedure

Infant–mother dyads were screened for eligibility (e.g., no complications during pregnancy or birth, full-term, singleton birth, birth weight > 2,500 g, no known health issues) during a telephone interview. Testing sessions were scheduled individually at a time when infants were expected to be in a calm and alert state. Upon arrival at the laboratory, the infant was given time to adapt to the environment while the experimenter explained the procedure and filled in a questionnaire about sleep, alertness and any deviations from routines on the testing day together with the mother. After giving written informed consent, each participating mother’s voice was recorded to create the individual familiar deviant for the oddball paradigm for each infant, respectively.

Electroencephalography (EEG), electrooculography (EOG), and electrocardiography (ECG) electrodes were applied while the mother distracted the infant or held the infant on her lap. After setting up the EEG recordings and making sure that the infant was in an alert and comfortable state (feeding or changing diaper beforehand, if necessary), the infant was positioned on a diaper changing unit lying down on its back with the head approximately in the middle between two loudspeakers (distance of approximately 45 cm, each). The mother stood in front of the unit and interacted with the infants (e.g., presenting hand puppets, blowing bubbles, changing facial expressions, etc.) to keep the infants calm and alert as has been suggested to be suitable in conducting infant studies (Brannon et al., 2008; Hoehl and Wahl, 2012). They were instructed not to talk to their child or make any kind of noises during the voice paradigm. If the infant was uncomfortable in this position, the mothers alternatively had their infant on their arm during recording, with the position of loudspeakers adjusted accordingly. The oddball paradigm was presented to the awake infant for 10 min and could be paused, if necessary, due to fussiness or changes in alertness (this was the case for two infants). Fifteen infants had slept upon arrival in the laboratory and most of them had been fed (*n* = 28) right before the presentation of the paradigm after the electrode placement.

### EEG Data Collection and Processing

Electroencephalography was recorded using soft Ag/Cl electrodes attached to an infant-suitable cap (EASYCAP GmbH, Herrsching, Germany) at electrode positions F3, Fz, F4, FCz, C3, Cz, C4, Pz, and mastoids, with reference to M2, and Fp2 as the ground. Electrode impedances were mostly below 10 kΩ, but always kept below 20 kΩ (which is considered acceptable for recording infant ERPs, Friedrich and Friederici, 2004; Hoehl and Wahl, 2012). Additionally, EOG recordings included one electrode below the left eye and one at the Fp1 position. Electrophysiological signals were digitized online at a rate of 500 Hz using a standard amplifier (BrainAmps, Brain Products GmbH, Gilching, Germany).

Offline, EEG data was processed using the Brain Vision Analyzer 2.0 Software (Brain Products GmbH, Gilching, Germany). The EEG was re-referenced to linked mastoids (in one participant, the signal from M1 was very noisy, therefore only M2 served as a reference) and filtered between 1 and 30 Hz and a 50 Hz notch filter (following Beauchemin et al., 2011). The signal was segmented into epochs of 1,000 ms from 200 ms pre-stimulus to 800 ms post-stimulus. ERP segments were rejected as artifacted in a channel-specific manner (Fujioka et al., 2011) when (1) a voltage difference >100 μV occurred within 300 ms, (2) a period of low activity (<0.5 μV) was detected for a period >100 ms, (3) absolute amplitudes exceeded ±100 μV, or (4) an ocular artifact was identified using a semiautomatic detection method (based on Independent Component Analysis, Hoffmann and Falkenstein, 2008). Baseline correction was applied using the 200 ms pre-stimulus period. Pz was excluded from further analyses as this channel contained too many artifacts to produce reliable trial numbers in the majority of participants, possibly due to the fact that infants were lying on the back of their heads.

To be included in analyses, electrodes were required to meet a criterion of 10 artifact-free epochs for familiar and unfamiliar deviants, respectively, and 50 artifact-free epochs for the standard stimuli (following recommendations for collecting infant ERP data, DeBoer et al., 2007; Hoehl and Wahl, 2012). This procedure resulted in participants having a differing number of contributing electrodes in their final frontal and central averages. The mean number of included epochs per participant across all electrodes was 278.9 (±17.5, range of 64–488) for the standard stimuli (corresponding to 54.7% of the total number of stimuli presented), 25.8 (±1.1, 10–45; 57.2%) for the familiar deviants and 25.8 (±0.8, 10–42; 57.3%) for the unfamiliar deviants.

### Data Analyses

**FIGURE 1:**
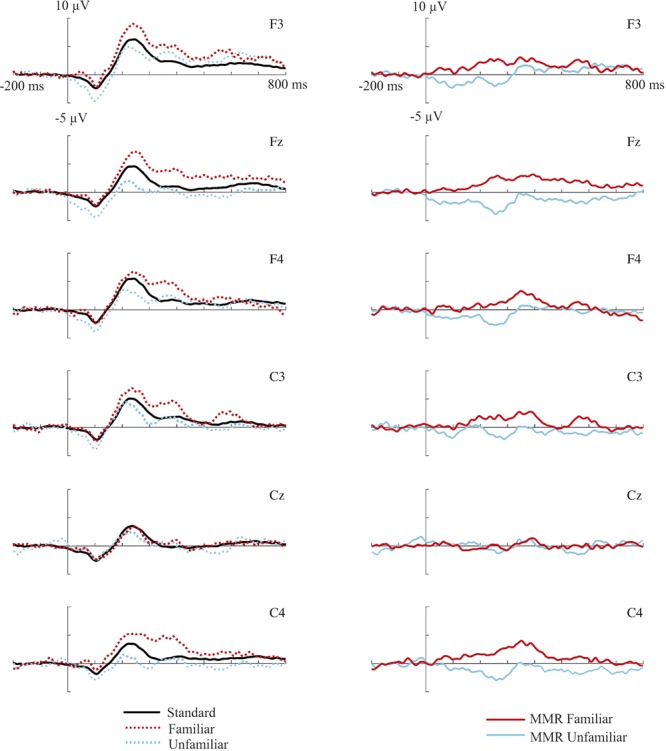
**(Left)** ERP responses to the frequent standard stimuli (black lines), familiar deviant stimuli (mother’s voice, dotted red/dark gray lines), unfamiliar deviant stimuli (stranger’s voice, dotted blue/light gray lines). **(Right)** MMR to the familiar (red/dark gray lines) and unfamiliar (blue/light gray lines) deviant voice stimuli (determined as difference wave forms by subtracting the ERP to standard stimuli). Mean potential responses recorded from the different frontal and central electrode sites are shown for an interval between –200 pre-stimulus onset to 800 ms post-stimulus onset.

First, epochs were averaged per condition (familiar and unfamiliar deviants, standard stimuli) for each participant, separately. The MMR was calculated as a difference wave by subtracting, per electrode site and participant, the ERP to the standard stimulus from the ERP to the unfamiliar deviant resulting in the MMR for the unfamiliar voice, and by subtracting the ERP to the standard stimulus from the ERP to the familiar deviant (mother’s voice) resulting in the MMR for the familiar deviant (see **Figure 1** for the mean ERP responses for each condition and the MMRs for each electrode, separately). Based both on visual inspection of the ERP data and findings from previous studies (e.g., Leppänen et al., 1997; Dehaene-Lambertz and Pena, 2001; Kushnerenko et al., 2002, 2007; Háden et al., 2009; see for a review, Kushnerenko et al., 2013) mean amplitude of the MMR was calculated for a 100–200 ms, a 200–300 ms and a 300–400 ms post-stimulus interval. To investigate possible differences between frontal and central electrode sites, F3, Fz, and F4 were averaged in one frontal average, C3, Cz, and C4 were averaged in a central average, for statistical analysis (see e.g., Partanen et al., 2013 for a similar approach of averaging over electrodes). To determine the intervals of a genuine MMR (significant difference between the response to the deviant compared to the standard stimuli), the original ERPs to all types of stimuli were compared in frontal and central electrode sites within the same post-stimulus intervals.

Results are reported as means (±SEM). Statistical analyses relied on repeated measures analyses of variance (ANOVA) with stimulus type, region (frontal vs. central) and post-stimulus interval as within-factors with *post hoc* tests for significant effects (Bonferroni corrected). Partial η^2^ was used to indicate central effect sizes. For the ANOVA, degrees of freedom were corrected using the Greenhouse–Geisser procedure where appropriate. A *p*-value < 0.05 was considered significant.

## Results

### Comparison of the Familiar and Unfamiliar MMRs

**FIGURE 2:**
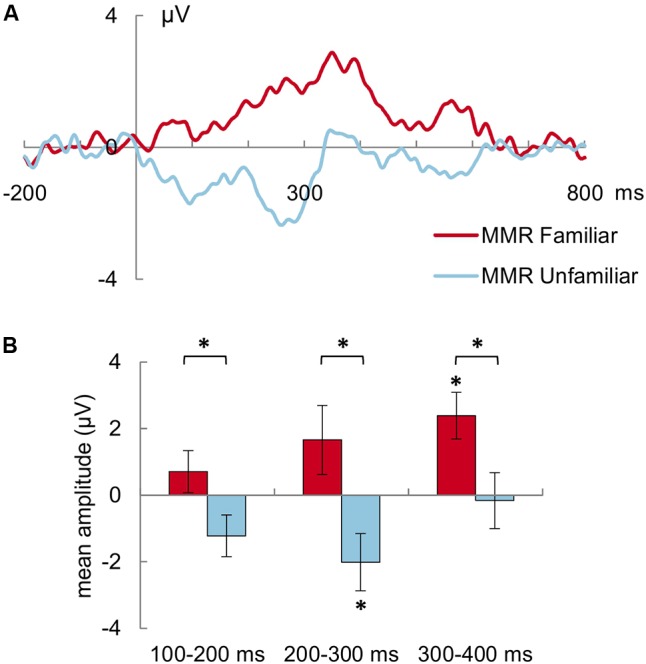
**(A)** Mismatch Responses (MMR) to the familiar (red/dark gray line) and unfamiliar voice (blue/light gray line) averaged across all frontal and central electrode sites and all subjects. (The MMR is determined by the difference between the ERPs to the familiar and unfamiliar deviant, respectively, minus the ERP to the standard voice stimuli.) The mean potential from -200 pre-stimulus onset to 800 ms post-stimulus onset is shown. **(B)** Mean (±SEM) amplitude of the MMR to the familiar (red/dark gray) and unfamiliar (blue/light gray) deviant voices for the 100–200 ms, 200–300 ms, and 300–400 ms latency ranges. Asterisk above brackets indicates *p* < 0.05 for pairwise comparison between MMR to familiar vs. unfamiliar deviants. Asterisk on top of SEM bar indicates significant (*p* < 0.05) MMR, i.e., a significant difference between the ERP to the deviant stimulus as compared to the ERP to the standard stimulus. The MMR to the familiar deviant is more positive than the MMR to the unfamiliar stimulus. Compared with the ERP to standard stimuli, the MMR to familiar deviants expresses itself as significant positive shift in the 300–400 ms post-stimulus interval, whereas the MMR to the unfamiliar deviants expresses itself as significant negative shift in the 200–300 ms post-stimulus interval.

The mean amplitude (in μV) of the MMR to the familiar deviant was more positive than the MMR to the unfamiliar deviant in general (MMR familiar: 1.58 ± 0.71 μV, MMR unfamiliar: -1.13 ± 0.68 μV; main effect of stimulus type: *F*(1,30) = 7.0, *p* = 0.013, $\eta_{p}^{2}$ = 0.19, **Figure 2**). This was also true when looking at the post-stimulus intervals separately: for 100–200 ms [*F*(1,30) = 6.0, *p* = 0.020, $\eta_{p}^{2}$ = 0.17], 200–300 ms [*F*(1,30) = 5.7, *p* = 0.024, $\eta_{p}^{2}$ = 0.16] as well as 300–400 ms [*F*(1,30) = 5.9, *p* = 0.022, $\eta_{p}^{2}$ = 0.16]. Additionally, the mean amplitude of the MMR was more positive in the 300–400 ms interval than the earlier two intervals in general [both *t*(30) > 2.7, *p* < 0.03; main effect of interval: *F*(2,60) = 5.3, *p* = 0.008, $\eta_{p}^{2}$ = 0.15].

### Comparison of the Original ERPs

Overall, the mean amplitude of ERP to the familiar deviant was more positive than the ERP to the standard stimuli (main effect of stimulus type: *F*(1.5,45.7) = 5.7, *p* = 0.011, *$\eta_{p}^{2}$* = 0.16). Importantly, the differences between the stimulus types seemed to depend on the post-stimulus interval [trendwise significant, interval × stimulus type interaction: *F*(2.7,81.1) = 2.7, *p* = 0.058, *$\eta_{p}^{2}$* = 0.08].

A significant difference between the ERPs to the familiar deviant and standard stimuli, i.e., a robust MMR, was only found in the 300–400 ms post-stimulus interval. In this interval, the mean amplitude of the ERP to the familiar deviant (3.84 ± 0.94 μV) was significantly larger, i.e., more positive, than the mean amplitude of the ERP to the standard stimuli [1.39 ± 0.62 μV, *t*(30) = 3.52, *p* = 0.002, *d*_z_ = 0.63]. No significant differences in mean amplitude of the ERPs to the familiar deviant and standard stimuli emerged for the 100–200 ms [*t*(30) = 1.13, *p* = 0.54] or the 200–300 ms post-stimulus interval [*t*(30) = 1.61, *p* = 0.24]. Looking at the ERP traces of single infants visually, 29 of the 31 infants descriptively showed some differentiation of the ERP to the familiar deviant from the ERP to the standard, 25 of those as a positive deflection.

A robust MMR differentiating between the ERPs to unfamiliar deviant and standard stimuli was only found in the 200–300 ms post-stimulus interval. In this interval, the mean amplitude of the ERP to the unfamiliar deviant (1.91 ± 0.96 μV) was smaller, i.e., more negative, than the mean amplitude of the ERP to the standard stimulus [3.96 ± 0.67 μV, *t*(30) = -2.36, *p* = 0.05, *d*_z_ = -0.42]. No significant differences in mean amplitude of the ERPs to the unfamiliar deviant and standard stimuli emerged for the 100–200 ms [*t*(30) = -1.95, *p* = 0.12] or the 300–400 ms post-stimulus interval [*t*(30) = -0.15, *p* = 1.0]. Descriptively, 26 of the 31 infants showed some differentiation of the ERP to the unfamiliar deviant from the ERP to the standard, 19 of those as a negative deflection.

Additionally, mean amplitudes were generally larger in the frontal electrodes compared to the central electrodes in the 200–300 [*t*(30) = 5.13, *p* < 0.001, *d*_z_ = 0.92] and 300–400 ms intervals [*t*(30) = 4.68, *p* < 0.001, *d*_z_ = 0.84] than the 100–200 ms interval [*t*(30) = 0.87, *p* = 0.39; interval × region interaction: *F*(2,60) = 13.4, *p* < 0.001, *$\eta_{p}^{2}$* = 0.31, main effect region: *F*(1,30) = 19.6, *p* < 0.001, *$\eta_{p}^{2}$* = 0.40; main effect interval: *F*(2,60) = 20.2, *p* < 0.001, *$\eta_{p}^{2}$* = 0.40].

### Control Variables

Infants were reported to have slept an average of 13.4 h (±0.4) in the last 24 h which is typical for infants of this age. No major deviations from daily routines were reported, other than two infants who received vaccinations 2 days before testing. For the subsample of infants where mothers were asked to rate their infant’s sleepiness, all infants scored ≤6 (2.8 ± 0.3), thus, not very sleepy. Correlational analyses of these control variables with the main dependent measures did not yield any indication that the mean amplitude measures were influenced by infants’ age, amount of sleep, or reported sleepiness (all *r* < 0.37, *p* > 0.08, uncorrected for multiple comparisons, there was a weak trend of higher sleepiness to correlated with lower values in the 100–200 ms interval for the familiar MMR and the familiar deviant ERP).

## Discussion

Our study in 3-month-old infants revealed a distinct MMR to unfamiliar and to familiar (mother’s voice) deviant stimuli – although this differential pattern has to be interpreted cautiously because of large variance – as well as a significant difference between these responses. Overall, the MMR (calculated as the difference between the ERPs to the deviants and the standard stimuli) was more positive for the familiar compared to the unfamiliar deviant across a large post-stimulus interval of 100–400 ms in frontal and central electrodes. For the familiar deviant (i.e., the mother’s voice), a robust MMR (with reference to the ERP to the standard stimuli) expressed itself as a significant positive deflection in the 300–400 ms post-stimulus interval. By contrast, for the unfamiliar deviant, a robust MMR (with reference to the ERP to the standard stimuli) expressed as a significant negative potential deflection in the 200–300 ms post-stimulus interval. Our findings confirm previous work in newborns (Beauchemin et al., 2011) showing that long-term memory-based processing of a familiar deviant voice is associated with a stronger positive MMR compared with responses to an unfamiliar deviant voice. The present study extends those findings in newborns in showing that, at the age of 3 months, processing of unfamiliar deviants is associated with a distinct negative MMR, occurring slightly earlier (200–300 ms) than the positive MMR to familiar deviant voice. Thus, our study shows a reliable dissociation between earlier and later components of the MMR in 3-month-olds, possibly reflecting short-term memory-based change detection processes and subsequent long-term memory-based processing of relevance, respectively.

We found a negative MMR to the unfamiliar deviant, robustly differentiating between the standard and the deviant in the 200–300 ms post-stimulus interval. This negative component probably reflects the early discrimination process that detects change in a stream of repeated stimuli maintained in short-term memory (Näätänen et al., 2007). Thus, it can be considered a precursor of the adult-like MMN. Also, the latency range in which this negative MMR is observed in the 3-month-olds roughly corresponds to that seen in adults, although in adults the MMN might occasionally overlap with the earlier N1 component (Näätänen et al., 2007; Duncan et al., 2009). The negative MMR to the unfamiliar deviant may suggest a more mature and adult-like response to the unfamiliar compared to the familiar stimuli. The reason why the negative component was not prominent in the MMR to the familiar deviant (although it clearly was discriminated) is probably the strong positive MMR in the subsequent 300–400 ms post-stimulus interval, which extends into the earlier interval. This suggests that the cognitive processes linked to the two MMR components are partly running in parallel.

In contrast to the negative MMR to the unfamiliar deviant, we found a positive MMR to the familiar deviant which robustly differentiated between the standard and the deviant in the 300–400 ms post-stimulus interval. This positive component likely reflects a recognition process that evaluates the relevance of the detected stimulus based on a comparison with long-term memory representations. It can be considered a precursor of the adult early P300 which has been related to a switch from automatic to attentional processing (e.g., Polich and Kok, 1995; Polich, 2007). This kind of relevance evaluation might be the basis of infants showing a preference for their own mother’s voice very early during ontogeny (DeCasper and Fifer, 1980; Lee and Kisilevsky, 2014). The existence of a long-term memory-modulated MMR component fits nicely with the notion that familiarity with a voice or phonemes of our native language, as well as trained auditory patterns, influence the magnitude and time course of change detection as reflected in the ERP response (Näätänen et al., 1997, 2007; Cheour et al., 1998, 2002; Atienza and Cantero, 2001; Tervaniemi et al., 2001). It is also in accordance with findings of the majority of MMR studies in newborns which show a frontocentral positive component peaking around 300 ms following deviant stimuli that are more or less familiar to the newborn (Leppänen et al., 1997; Dehaene-Lambertz and Pena, 2001; Winkler et al., 2003). Although our findings are in line with past research, influences of multi-sensory integration may also be present. Specifically, mothers could be seen during the task while the females providing the standard and unfamiliar deviant voices were not present. Nevertheless, influences of multi-sensory integration should be minimal, as mothers did not actually speak during the task, therefore no visual stimulus of the mother’s mouth moving was present for the infant to integrate with the sound of the familiar deviant. Furthermore, we cannot directly measure if infants recognized their mother’s voice. However, given that infants have previous experience with their mother’s voice in unemotional, non-infant directed settings and processing of the voice recordings was kept to a minimum this seems unlikely. Taken together, our findings add evidence to the notion that the polarity of the MMR during infancy is highly sensitive to the familiarity of the deviant stimuli, with unfamiliar deviants producing an earlier negative potential deflection and familiar deviants producing a later positive potential deflection (Hirasawa et al., 2002; Martynova et al., 2003; Cheour, 2007; Beauchemin et al., 2011).

Our findings corroborate the only study thus far that has investigated the influence of familiarity on the MMR in newborns and showed that the MMR to a familiar deviant is more positive than to an unfamiliar one (Beauchemin et al., 2011). In comparison with those findings in newborns where the effect of a more positive MMR to the familiar than unfamiliar deviant concentrated on an early (around 200 ms) and late (around 500 ms) post-stimulus interval, in our 3-month-olds the effect starts earlier and is robustly observed over a broader 100–400 ms post-stimulus interval. These differences might partly reflect maturational changes, i.e., shortening of latencies and changes in scalp distribution over the course of infancy and childhood (Jing and Benasich, 2006; Cheour, 2007). Furthermore, our 3-month-olds show a reliable early MMR of negative polarity to the unfamiliar voice, which was not present in the newborns. The newborns, instead, showed a late positive MMR also to the unfamiliar deviant (with reference to the standard stimuli) which reached significance only after 400 ms. The emergence of an early negative MMR to the unfamiliar deviant in our 3-month-olds, thus, might reflect the emergence of effective short-term memory-based processing of stimulus deviation and is in accordance with longitudinal studies showing that a robust negative MMR starts to appear only after a couple of months (Kushnerenko et al., 2002; Jing and Benasich, 2006; He et al., 2009). In light of the negative polarity of this MMR component, the mechanisms likely differ in quality from those associated with the late positive MMR which in the 3-month-olds (unlike in newborns) appeared to be exclusively related to long-term memory-based comparison processes. The expression of a negative MMR to unfamiliar deviants reminiscent of the MMN in adults in our 3-month-olds might be related to the relatively fast maturation of the auditory cortex during the first months of life (Huttenlocher and Dabholkar, 1997) and also to experience with voices in general and familiar voices in particular during this period. However, it has to be noted that conclusions drawn from the comparison between the present study in 3-month-olds and Beauchemin et al.’s study in newborns remain tentative as the experimental procedures were not exactly the same. Thus, we used longer word stimuli instead of a single vowel and also our babies were awake whereas in the Beauchemin et al.-study the babies were in active sleep during stimulus presentations. These factors *per se* might have systematically changed MMRs in the two studies (Friederici et al., 2002; Otte et al., 2013; but see Cheour-Luhtanen et al., 1996 for no influence of alertness level on the MMR).

Using the MMR as an electrophysiological marker in a voice discrimination paradigm, we reliably dissociated components linked to short-term memory-based from long-term memory-based processing in 3-month-old infants. A robust negative MMR to an unfamiliar deviant in the 200–300 ms post-stimulus interval possibly reflects an earlier change detection process. On the contrary, a robust positive MMR to the familiar deviant (mother’s voice) might reflect processing of the relevance of the input utilizing long-term memory representations. Our study, thereby, extends previous findings in newborns showing a robust differentiation between familiar (mother’s voice) and unfamiliar deviants with a more refined dissociation between change detection (short-term) and relevance processing (modulation by long-term representations) in 3-month-old infants.

## Author Contributions

KZ and JB designed the study. KZ, EB, and JB wrote the manuscript. KZ and LT conducted the study and analyzed the data.

## Conflict of Interest Statement

The authors declare that the research was conducted in the absence of any commercial or financial relationships that could be construed as a potential conflict of interest.
